# Progress risk assessment of oral premalignant lesions with saliva miRNA analysis

**DOI:** 10.1186/1471-2407-13-129

**Published:** 2013-03-19

**Authors:** Ya Yang, Yue-xiu Li, Xi Yang, Long Jiang, Zuo-jun Zhou, Ya-qin Zhu

**Affiliations:** 1 Department of General Dentistry, Ninth People’s Hospital, School of Stomatology, Shanghai Jiao Tong University School of Medicine,Shanghai Key Laboratory of Stomatology, 639 Zhi Zao Ju Road, Shanghai, 200011, China; 2 Department of Stomatology, Tai’an Central Hospital, Tai’an, Shandong, 271000, China; 3 Department of Oral & Maxillofacial-Head & Neck Oncology, Ninth People’s Hospital, School of Stomatology, Shanghai Jiao Tong University School of Medicine, Shanghai Key Laboratory of Stomatology and Shanghai Research Institute of Stomatology, Shanghai, 200011, China

**Keywords:** Oral leukoplakia, Malignant transformation, Risk assessment, miRNAs, Salivary biomarker

## Abstract

**Background:**

Oral cancer develops through multi-stages: from normal to mild (low grade) dysplasia (LGD), moderate dysplasia, and severe (high grade) dysplasia (HGD), to carcinoma *in situ* (CIS) and finally invasive oral squamous cell carcinomas (OSCC). Clinical and histological assessments are not reliable in predicting which precursor lesions will progress. The aim of this study was to assess the potential of a noninvasive approach to assess progress risk of oral precancerous lesions.

**Methods:**

**W**e first used microRNA microarray to profile progressing LGD oral premaligant lesions (OPLs) from non-progressing LGD OPLs in order to explore the possible microRNAs deregulated in low grade OPLs which later progressed to HGD or OSCC. We then used RT-qPCR to detect miRNA targets from the microarray results in saliva samples of these patients.

**Results:**

We identified a specific miRNA signature that is aberrantly expressed in progressing oral LGD leukoplakias. Similar expression patterns were detected in saliva samples from these patients.

**Conclusions:**

These results show promise for using saliva miRNA signature for monitoring of cancer precursor lesions and early detection of disease progression.

## Background

Oral squamous cell carcinomas (OSCCs) are among the most common types of head and neck cancers and are a major cause of significant morbidity. It was reported that 16–62% of OSCCs develop from premalignant lesions [1], which often presents clinically as white or red mucosal patches called leukoplakia and erythroplakia. Early detection of cancer development from oral premalignant lesions (OPLs) plays a crucial role in successful therapy. Currently risk of progression in oral leukoplakia is typically determined based on clinical assessment and histopathological evaluation of biopsied material. High grade dysplasia (HGD) and carcinoma *in situ* (CIS) are considered to have a high risk for progression to invasive disease. In contrast, most of the low grade dysplasias (LGDs) remain unchanged for years or even resolve over time [2]. But a small proportion of these LGDs may progress to carcinomas [3]. Clinical and histological characteristics cannot be used to separate “progressing” and “non-progressing” LGDs [4]. There is therefore an urgent need to find predictive biomarkers that can aid in defining progression likelihood of LGDs, which represent the majority of diagnosed OPLs.

miRNAs are an abundant class of small 18–25 nucleotides long single-stranded non-coding RNA. These non-coding RNAs participate in a variety of biologic processes including development, differentiation, apoptosis and proliferation through regulating its target genes [5-8]. Notably, a single miRNA is capable of regulating the translation of a multitude of genes [9]. And they are remarkably stable both in saliva samples and in tissue samples [10,11], which offers a great advantage over other classes of biomarkers and also an extremely important characteristic in clinical settings. The control of gene expression by miRNAs is a process seen in virtually all cancer cells. Recently, a bundle of studies have showed that miRNAs might behave as cancer ‘drivers’ and the aberrant expressions could profoundly contribute to the progression from premalignant lesion to cancer [12-14]. Only a small number of studies have investigated miRNA expression profiles in the malignant transformation of OPLs, and there is no report yet about investigating salivary miRNAs changes in this type of patients. Cervigne *et al.* examined miRNA expression changes in tissue samples during progression of oral malignancy [15]. Wiklund *et al.* demonstrates that Certain OSCC specific miRNA are both present and detectable in oral fluids [16]. However salivary miRNA changes involved in the transformation of oral leukoplakias remain poorly understood.

With an aim to investigate the potential of using salivary microRNA biomarkers to assess the risk of malignant transformation, our study includes the analysis of progressing and non-progressing LGD leukoplakias with similar histopathological diagnosis but different clinical outcomes. We first used microRNA microarray to profile progressing LGDs from non-progressing LGDs. We identified a specific miRNA signature that is aberrantly expressed in oral LGD leukoplakias which later progressed to HGDs or CISs and even OSCCs. Similar miRNA deregulation patterns were detected in saliva samples of these patients. These results indicated the feasibility of a noninvasive assay for risk assessment of oral precancerous lesions based on salivary miRNA signatures.

## Methods

### Patients and samples collection

**Figure 1:**
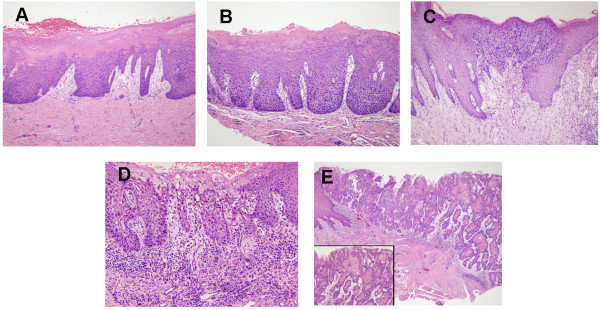
**Representative histological pictures of OPLs and OSCCs.** (**A**) oral leukoplakia with low grade epithelia dysplasia; (**B**) oral leukoplakia with moderate epithelia dysplasia; (**C**) oral leukoplakia with high grade epithelia dysplasia; (**D**) cancerated leukoplakia; (**E**) invasive oral squamous cell carcinoma.

From 2006 to 2012, 45 eligible patients treated in the department of Oral & Maxillofacial-Head & Neck Oncology, Ninth People’s Hospital, Shanghai Jiao Tong University (Shanghai, China) were enrolled in the present study. The patients had been diagnosed with LGD leukoplakias and were given 13-*cis*-retinoic acid (13cRA) treatment and close follow-ups before any event (defined as the diagnosis of HGD or CIS or OSCC). The diagnosis of leukoplakias, OSCC, and LGD were made according to World Health Organization criteria [17]. A total of 45 frozen samples were collected at baseline after enrollment. Among these 45 LGD leukoplakias patients, 10 patients progressed to carcinoma in situ or OSCC after an average time of 30.1 months. 2 were excluded because of poor RNA quality. Among the 35 patients who did not develop OSCC, 5 were excluded because of poor RNA quality, and another 12 were excluded because of new lesion occurrence. As these progressing LGD samples are very rare and difficult to obtain, we included all the 8 samples from patients who developed oral cancer (N = 8) for gene expression profiling. 7 patients who remained at a LGD stage within 3–5 years of follow-up were selected, in order to identify the differences in miRNA expression between these precancerous lesions with different clinical outcomes. Informed consent was obtained from all patients. The project was approved by the Scientific and Ethics Committee of Shanghai Jiao Tong University. Patient information and representative histological pictures is shown in Table 1 and Figure 1, respectively.

**Table 1:** Sample set of oral LGD leukoplakias

		**Histopathological**			**Tobacco**	**Duration of**
**Patients**	**Lesion site**	**Diagnosis**	**Age**	**Sex**	**Exposure**	**follow-up(mo)**
1	Left lateral tongue	Mild to moderate dysplasia	45	female	former smoker	40
2	Right lateral palatine	Mild to moderate dysplasia	67	male	current smoker	36
3	Right lateral palatine	Mild to moderate dysplasia	55	female	never smoked	39
4	right lateral tongue	Mild to moderate dysplasia	54	female	never smoked	36
5	right cheek mucosa	Mild to moderate dysplasia	64	male	current smoker	43
6	right cheek mocusa	Mild to moderate dysplasia	57	male	current smoker	46
7	left cheek mucosa	Mild dysplasia	61	male	current smoker	39
8	left lateral tongue	Severe dysplasia SCC micro-foci	71	male	current smoker	29
9	lower lip	Severe dysplasia, SCC micro-foci	60	female	never smoked	28
10	left cheek	Severe dysplasia SCC micro-foci	52	female	former smoker	34
11	palate	Severe dysplasia	55	female	never smoked	35
12	left cheek	Severe dysplasia	66	male	former smoker	30
13	right lateral tongue	carcinoma in situ	61	female	never smoked	29
14	right lateral tongue	carcinoma in situ	71	male	current smoke	30
15	left cheek	moderate to severe dysplasia SCC micro-foci	59	male	current smoker	26

Saliva samples were collected from these patients whenever tissue biopsies were collected. But only samples collected at first visit were used for analysis in order to investigate miRNA expression differences in these premalignant lesions at LGD stage. Saliva samples from 7 healthy volunteers were also collected to determine baseline miRNA expression in oral saliva from normal control. Briefly, immediately after mouthwash, ~2 ml saliva was collected. Upon collection, saliva samples were mixed with 5 ml RNA Protect Saliva reagent (Qiagen, Copenhagen), kept at room temperature for 24 hours, and then stored at −20°C until RNA or protein isolation.

### RNA extraction, cDNA synthesis and miRNA expression profiling for tissue samples

The TaqMan^®^ low density array (TLDA) qRT-PCR system (Applied Biosystems, Foster City CA) were used for global miR expression analysis. Briefly, total RNA was extracted using TRIzol reagent (Invitrogen).The qualities of RNA extracts were found to be good. Total RNA was converted into specific cDNA derived from mature miRs and quantified using Multiplex RT and TaqMan Low Density Array (TLDA) Human MicroRNA Panel v3.0 and 7900 real-time RT PCR System (Applied Biosystems). A total of 800 ng RNAs were used for each array. The two-card set of TaqMan^®^ Array MicroRNA Cards (Cards A and B) contained 754 lyophilized human TaqMan miR sequences. And each card contains four control assays—three carefully selected candidate endogenous control assays and one negative control assay.

### RNA extraction, cDNA synthesis and miRNA quantification for saliva samples

Real-time qPCR analysis was performed on saliva samples from these patients and 7 healthy volunteers using the TaqMan MicroRNA Assay. miRNA expression analysis was performed for 8 selected deregulated miRNAs (miR-10b, miR-145, miR-99b, miR-708, miR-181c, miR-30e, miR-660 and miR-197) with greatest fold changes in progressing LGD leukoplakias compared with non-progressing LGD leukoplakias.

Total RNA was extracted from saliva samples using the RNeasy Micro Kit (Qiagen, Hilden, Germany) according to the manufacturers’ protocols. In brief, the saliva sample is first centrifuged, and Buffer RLT is then added to the resulting pellet. After addition of ethanol, the sample is applied to an RNeasy MinElute spin column, where RNA binds to the membrane after centrifugation. Traces of genomic DNA are removed by DNase digestion, and contaminants are washed away in several wash steps. Highly pure RNA is then eluted using RNase-free water in a volume of just 14 μl. The purity of isolated RNA was determined by OD260/280 using a Nanodrop ND-1000 (Thermo Scientific, Worcester, MA). Each RNA sample was polyadenylated and reversely transcribed to cDNA using the TaqMan^®^ MicroRNA Reverse Transcription Kit (Applied Biosystems, Foster City, CA Single-stranded cDNA was synthesized from 10 ng of total RNA(normalization by amount) or from 1 μl of the RNA volume (normalization by volume) using specific miRNA primers (TaqMan MicroRNA Assay, PN 4427975, Applied Biosystems).

All assays were performed in duplicate. Expression data were normalized against RNU6; pooled saliva samples from healthy volunteers were used as the reference for analysis.

### Statistical analyses

Samples were grouped as normal, non-progressive LGD leukoplakia and progressive LGD leukoplakia. Data were quantified and analyzed using Sequence Detection System (version 2.3) (Applied Biosystems). The expression level of each miRNA was quantified by its normalized threshold cycle number ΔCt, where ΔCt = [Ct (miRNA)]-[Ct (U48)], and the relative expression level was calculated as 2^-(ΔCt)^ which is commonly used in genom-wide profiling studies of miRNAs. In order to select differentially expressed miRNAs for further classification, the random variance t-test (RVM t-test) was used to compare the control and experiment, as the RVM t-test can raise degrees of freedom effectively in the cases of small samples. The multiple testing was corrected using the Benjamini–Hochberg false discovery rate (FDR) method. Having a differential fold change > ±2 at raw *P* value < 0.05 were considered as significant candidates.

Expression levels of selected miRNAs in saliva samples of these patients were calculated with U6 as normalization factor. Comparison of raw U6 values between different groups showed no significant difference (p = 0.09, Mann–Whitney *U* test). Raw data were normalized by subtracting the U6 CT values from the marker CT values. Statistical comparisons were made with the use of student’s t-tests for two groups. Data analyses were performed using SPSS for Windows version 16.0 (SPSS Inc., USA). Tests were two-sided and p ≤ 0.05 was considered statistically significant.

## Results

### miRNA expression profiling in different types of LGD lesions

In order to evaluate miRNA deregulations of progressive oral premalignant lesions, we used a systematic miRNA expression profiling analysis of 754 mammalian miRNAs on 8 progressing LGD leukoplakias and 7 non-progressing LGD leukoplakias. A filter procedure reduced the number of miRNAs to a total of 617 hsa-miRNAs, eliminating genes with low expression variation across the experiments (SD < 0.3). We performed a differential expression analysis to detect significant differences in miRNA expression between different types of LGD leukoplakias.

**Figure 2:**
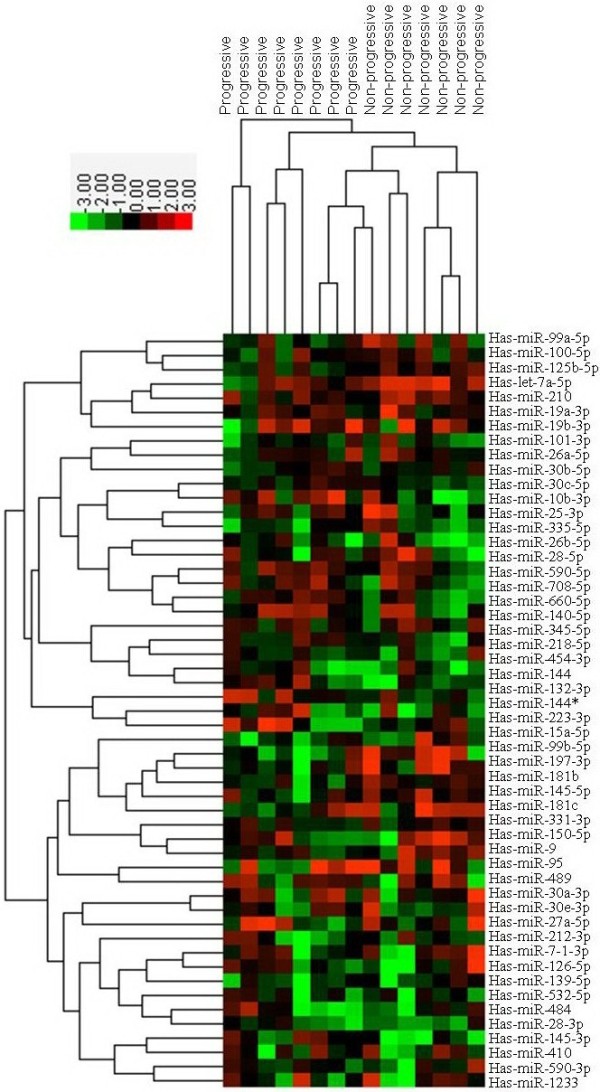
**Differentially expressed miRNAs between progressive LGD leukoplakias and non-progressive LGD leukoplakias.** Heat map showing the differentially expressed mirRNAs between progressing LGDs and non-progressing LGDs. Over-expressed miRNAs are colour-coded red, repressed miRNAs are colour-coded green.

We identified 25 miRNAs differentially expressed between progressive and non-progressive LGD leukoplakias. Compared to non-progressive LGD leukoplakias, 13 miRNAs were down-regulated and 12 miRNAs were up-regulated in progressive LGD leukoplakias. According to previous studies on oral squamous cell carcinomas, most of these deregulated miRNAs in progressive LGD leukoplakias were changed with same changing patterns as that in OSCC, such as down-regulation of miR-99, miR-let-7, miR-145 and up-regulation of miR-708, miR-10b, miR-26a and miR-30e. Interestingly, in our study both miR-181c and miR-181b were found to be under-expressed in progressing LGD leukoplakias, which was contrary to the result of a previous study on miRNA expression changes during progression of oral premalignancy. The miRNAs that were differentially expressed are listed in Table 2 and Figure 2.

**Table 2:** miRNAs differentially expressed between progressing-LGD leukoplakias and non-progressing-LGD leukoplakias

	**Mean intensity in**	**Mean intensity in**			
**miRNA**	**Non-progressing LGDs**	**Progressing LGDs**	**Fold change**	**Unadjusted p value**	**FDR**
miR-10b-5p	10.2689	13.7939	11.51↑	0.008	0.069
miR-99a-5p	10.9423	8.1768	6.80↓	0.011	0.069
miR-99b-5p	16.3981	12.1692	18.75↓	0.003	0.016
miR-145-5p	13.3692	9.6968	12.75↓	0.011	0.069
miR-100-5p	11.9107	8.8512	8.33↓	0.011	0.0698
miR-125b-5p	10.9423	8.1768	6.80↓	0.011	0.0698
miR-181b	9.9163	6.1030	14.05↓	0.016	0.069
miR-181c	20.6273	16.6825	15.40↓	0.011	0.069
miR-197-3p	16.4423	12.3639	16.89↓	0.042	0.128
miR-331-3p	10.9681	8.1938	6.84↓	0.010	0.069
miR-15a-5p	16.5175	12.8715	12.51↓	0.002	0.056
miR-708	8.8610	12.4334	11.89↑	0.014	0.069
miR-150-5p	7.9801	5.4854	5.63↓	0.002	0.0567
miR-30e-3p	6.6441	10.9956	20.41↑	0.002	0.056
miR-30a-3p	15.2221	17.1464	3.80↑	0.049	0.985
miR-21	7.1456	9.5243	5.20↑	0.049	0.985
let-7a-5p	20.3130	17.7931	5.74↓	0.039	0.985
miR-335-5p	9.2299	10.2837	2.08↑	0.039	0.995
miR-144*	11.5283	15.1400	12.22↑	0.005	0.057
miR-25-3p	8.8177	12.5057	12.88↑	0 .017	0.069
miR-19a-3p	5.3255	8.5336	9.24↑	0.007	0.066
miR-660-5p	7.8916	12.6950	27.92↑	0.020	0.076
miR-140-5p	11.1943	15.2157	16.23↑	0.012	0.069
miR-590-5p	7.4825	11.4638	15.79↑	0.004	0.057
miR-9	21.1533	17.9955	8.92↓	0.039	0.915

### Detection of deregulated miRNA expressions in saliva

To explore the possibility of using salivary microRNA markers to distinguish OPL patients with different malignant transform potential, we tested the expression of 8 miRNAs (miR-10b, miR-145, miR-99b, miR-708, miR-181c, miR-30e, miR-660 and miR-197) in saliva samples from these patients using qRT-PCR. According to the TLDA findings, miR-10b, miR-660, miR-708 and miR-30e demonstrated significant over-expression in progressive LGD leukoplakias; miR-145, miR-99b, miR-181c and miR-197 were under-expressed in this group of leukoplakias.

**Figure 3:**
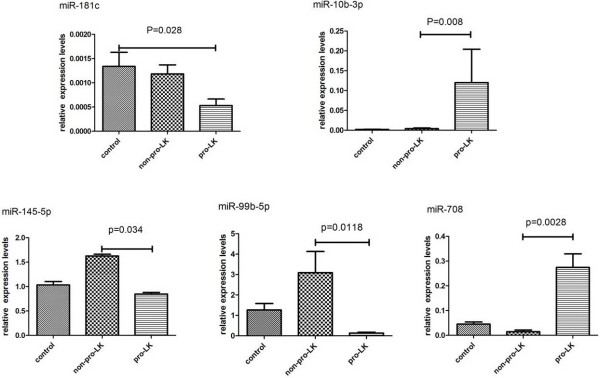
**Detection of miRNA targets by qRT-PCR in saliva samples from patients with progressing and non-progressing LGD leukoplakias as well as healthy volunteers.** Differences were estimated by t-test and p values are shown for each comparison.

Expressions of miR-10b, miR-145, miR-99b, miR-708 and miR-181c were significantly different in saliva of progressive LGD leukoplakia patients, compared to that of non-progressive LGD leukoplakia patients (Figure 3). Trend (P < 0.10) were observed for miR-197 and miR-30e, the p-values were 0.057 and 0.089 respectively. Expression of miR-660 was under-expressed in non-progressing LGD leukoplakias (p = 0.038) but over-expression of this miRNA in progressing leukoplakias did not reach statistical significance (P = 0.098), which was inconsistent with our initial TLDA findings of this gene in progressing leukoplakias.

## Discussion

Patients with oral premalignant changes consist of a diverse population and should be treated differently depending on their molecular genotype. There is an urgent need to identify which leukoplakia will progress and which will not. The search for genetic biomarkers of tumor progression is one of the potential approaches. miRNA expression profiling has been able to identify signatures associated with cancer initiation and progression. And abnormal changes in miRNA expression have been identified in patients’ bodily fluids, such as blood, urine, and saliva [18,19]. In order to explore the possibility of salivary miRNA analysis as a method aiding in risk assessment of OPL, we examined miRNA expression in paired tissue and saliva samples from patients with histopathologically similar leukoplakias but different clinical outcomes. We successfully detected in OPL patients’ saliva samples those miRNAs which were differentially expressed in OPL tissue samples. We found that saliva contains these deregulated miRNAs in much lower amounts, but they are present in this fluid and the changing patterns are similar with that in tissue samples.

In the present study we observed up-regulations of miR-708, miR-10b, miR-19a, miR-30e, miR-26a, miR-660 and down-regulations of miR-99, miR-15a, miR-197, miR-145 and miR-150. The same changing patterns have been reported in other cancers, including oral squamous cell carcinomas. However, we noticed miR-181c and miR-181b were found to be under-expressed in progressing LGD leukoplakias and over-expressed in non-progressive LGD leukoplakias, which was contrary to the result of a previous study. The miR-181 family members were identified as either tumor suppressors or oncogenes in various cancers depending on tissue types [20-22]. Cervigne N.K. et al. reported that they found a gradual increase in expression of miR-181b in tissue samples during oral carcinogenesis, suggesting miR-181b plays as an oncogenic gene in oral cancer [15]. Our results were contrary to this but in concordance with results of some recent studies. For example, decreased serum miR-181a was found in breast cancer patients, especially early stage breast cancer patients (ductal carcinoma in situ, TNM I and II) [23]. Visone R. et al. found that during the course of the disease the expression of miR-181b was decreased in samples of patients with a progressive but not in samples of patients with a stable disease. It seems that miR-181 family members may act as tumor suppressors in tumor initiation and decreased expression of these genes may contribute to progression from precursor lesions to tumors. Further studies needed to clarify this point.

We also noticed that a group of miRNAs are over-expressed in non-progressing LGD leukoplakias compared with progressing LGD leukoplakias. Among them are miR-197, miR-let-7, miR-99a/b, miR-126 and miR-145. These over-expressed miRNAs are known for their tumor suppressive roles in cancers. The marked up-regulation of these miRNAs in this group of patients implies that these stable LGD lesions may possess some mechanisms of protection from malignant transformation.

Several important conclusions can be made from the encouraging results of our study. Firstly, some miRNAs are differentially expressed in LGD leukoplakias with different progression potentials. Secondly, these differentially expressed miRNAs can be detected in both tissue samples and saliva samples as early as at LGD stage. Thirdly, Salivary miRNA analysis is a promising non-invasive assay for early detection and monitoring of oral premalignancies. But it should also be noted that our results were drawn from a small number of patients, as these progressing LGD leukoplakias are rare and very difficult to obtain. The different microRNA patterns are not intended to draw any disease specific conclusions and shown only as a proof of concept. However, when we compared the miRNA expression profiles of these LGD leukoplakias with different clinical outcomes, we did demonstrate a clear segregation between them and the similar expressions were detected in saliva samples of these patients. These preliminary results are suggestive to further develop this approach and the actual clinical value of this approach needs to be approved in larger cohort studies in high-risk groups.

## Conclusion

Right now biopsy should still remain the gold standard in diagnosing oral precancer and oral cancer. However, detection of deregulated miRNA biomarkers in saliva samples is a promising noninvasive assay for risk assessment of LGD oral precancerous lesions, which may add value to the histologic diagnosis.

## Abbreviations

HGD: High grade dysplasia; LGD: Low grade dysplasia; CIS: Carcinoma *in situ*; OSCC: Oral squamous cell carcinomas; OPLs: Oral premaligant lesions; FDR: False discovery rate; TLDA: TaqMan^®^ low density array.

## Competing interests

The authors declare that they have no competing interests.

## Authors’ contributions

YY and YZ were responsible for the study design, interpretation of the data and revision of the manuscript. YL and XY collected and processed the patients’ samples, YY, LJ and ZZ carried out the experimental work the statistical analysis. YY prepared the manuscript, and YZ made critical revisions. All authors read and approved of the final manuscript.

## Pre-publication history

The pre-publication history for this paper can be accessed here:

http://www.biomedcentral.com/1471-2407/13/129/prepub
